# CD4 T cell phenotype after AS01-adjuvanted immunization is shaped by prior antigen or pathogen exposure

**DOI:** 10.3389/fimmu.2026.1800411

**Published:** 2026-06-22

**Authors:** Robbert G. van der Most, Geert Leroux-Roels

**Affiliations:** 1 VaxxCellence, Antwerp, Belgium; 2 Center for Vaccinology (CEVAC), Ghent University and Ghent University Hospital, Ghent, Belgium

**Keywords:** adjuvant, AS01, CD4 T cells, cytokine expression, vaccine, IGN-g, human, immune memory

## Abstract

CD4 T-cell responses are important for the protection conferred by many infectious disease vaccines and can be quantitatively and qualitatively improved by adjuvants. The AS01 adjuvant is present in critical licensed or candidate vaccines against, amongst others, zoster, respiratory syncytial virus, malaria and tuberculosis. During their clinical development, these vaccines have been evaluated in populations ranging from immunologically naïve to strongly pathogen-primed individuals. We dissected the vaccine-induced CD4 T-cell responses across these studies, to elucidate how pre-existing immune memory in the host drives the polyfunctional phenotype of the vaccine-induced CD4 T cells. Across the investigated vaccines, a consistent pattern emerged, in which the capacity of antigen-specific vaccine-induced CD4 T cells to produce interferon-γ appeared to depend on increased levels of prior exposure, leading to pre-existing memory T cells. As the dominant origin of a vaccine-induced CD4 T-cell response (i.e., memory cells with a pre-existing T-cell receptor repertoire, or newly recruited naïve cells) remains to be clarified, this observation helps to guide future research into efficacious next-generation vaccines tailored to the specific immune priming status of the target population.

## Introduction

Emerging pathogens and global demographic trends drive the demands for effective vaccines. While current correlates of protection are mostly based on functional antibodies (1), for many diseases, protective immunity relies on both serological and cell-mediated immune (CMI) responses. Particularly CD4 T cells take center stage, given that T follicular helper (T_FH_) cells shape the antibody response quantity and quality (2), and that different CD4 effector T cells such as the T helper (Th)1, Th2 or Th17 phenotypes direct pathogen-specific immune effector responses. For example, CD4 T-cell memory may be important for protective immunity against viral and parasitic diseases, such as human immunodeficiency virus 1 (HIV-1), herpes zoster, respiratory syncytial virus (RSV), or malaria, as well as intracellular pathogens such as *Mycobacterium tuberculosis* (Mtb), the causative agent of tuberculosis (TB) (3–7).

Immune responses to recombinant protein-based antigens can be improved by vaccine adjuvants (8). This review focuses on AS01, an adjuvant containing the Toll-like-receptor (TLR)-4 agonist 3-*O*-desacyl-4′-monophosphoryl lipid A (MPL), the saponin QS-21 (*Quillaja saponaria* Molina, fraction 21) and liposomes (9). The selection of this adjuvant was informed by (i) the fact that AS01 has been shown to enhance antigen-specific polyfunctional CD4 T cell-mediated immunity; and (ii) the availability of human CMI data for a variety of AS01-adjuvanted vaccines and host immune backgrounds. The innate immune responses induced by AS01-adjuvanted hepatitis B surface antigen (HBs), mainly comprising interferon (IFN)-related transcriptional and cytokine responses, were shown to correlate with the antibody response quality/quantity as well as with the magnitude of the CD4 T-cell response (10–13). AS01 is included in several licensed vaccines (9), such as the recombinant zoster vaccine (RZV)/AS01 which contains the varicella-zoster virus glycoprotein E (VZV gE) antigen; the RSVPreF3/AS01 vaccine containing recombinant RSV glycoprotein F stabilized in the pre-fusion conformation; and the malaria vaccine RTS,S/AS01, containing the circumsporozoite (CSP) malaria antigen presented on an HBs carrier. The TB vaccine candidate M72/AS01, containing a fusion protein derived from recombinant Mtb32A and Mtb39A, is currently undergoing Phase III evaluation (NCT06062238). Finally, AS01 has been included in investigational HBs vaccines (8) and in two HIV-1 candidate vaccines, namely gp120-NefTat/AS01, containing the HIV envelope glycoprotein, regulatory protein Nef and transactivator of transcription Tat (14), and F4/AS01, containing a recombinant fusion protein encoding the HIV-1 clade B antigens p24, reverse transcriptase, Nef and p17 (15). Target populations varied across naïve individuals, such as the pediatric RTS,S/AS01 vaccinees or naïve adult trial participants, to primed individuals, comprising most adult RZV/AS01 and RSVPreF3/AS01 vaccinees also including immunocompromised (IC) individuals (16–19). Infants, older adults, and IC individuals such as hematopoietic cell transplantation (HCT) recipients, exhibit a reduced ability to mount polyfunctional CD4 T-cell responses to vaccination (20, 21).

Beyond age and immunocompetence status, pre-existing immune memory (reflecting prior infection or vaccination history) can also shape the quantity/quality of the CD4 T-cell response (22–24). The impact of a variety of background parameters, such as age, sex, immunocompetence, microbiome and others on vaccine responsiveness has been described elsewhere (25–28). However, exploring this critical nuance was considered beyond the scope for the current review, which focuses on the relationships between previous pathogen and vaccine priming and CD4 phenotypes. Indeed, while innate immunity characterizations were supported by single-cell RNA sequencing (scRNA-seq) assays, as reported for HBs/AS01 (13), only few scRNA-seq datasets involve vaccine-induced CD4 T-cell responses. One of these datasets, interrogating RZV/AS01-induced CD4 T cells in older adults using human leukocyte antigen (HLA)-II tetramers (29), showed that (i) CD4 T cells display a peak activation gene signature at 14 days after each vaccination; (ii) after a year, this activation gene signature did not return to baseline (leading the authors to argue that CD4 memory cells “retain markers of cell activation one year following vaccination”); and (iii) baseline T cell receptor (TCR) clonotypes still dominated the repertoire after a year, suggesting that the vaccine engages mostly cells with the pre-existing TCR repertoire. The latter proposal contrasts with observations suggesting that RZV/AS01 predominantly recruits naïve CD4 T cells (30). These differences notwithstanding, the contrasting data underscore the need for similar data for RZV/AS01 and other vaccines, involving larger sample sizes and different cell phenotypes and study populations, to gain deeper mechanistic insights into the origin of the CD4 T-cell response to vaccines.

Many of the clinical studies of AS01-adjuvanted vaccines evaluated changes in the quantity and polyfunctionality of antigen-specific CD4 T cells, using validated 7- or 9-color intracellular cytokine staining (ICS) assays. Peripheral blood mononuclear cells (PBMC) or whole blood samples stimulated *in vitro* with peptide pools (31, 32) yielded statistically concordant results (31). Antibody cocktails were specific to CD3, CD4, CD8, and the expression markers to CD40L, interleukin (IL)-2, IFN-γ and tumor necrosis factor (TNF), and, in some studies (33–35), also IL-13 and IL-17, allowing comparison of the phenotypes of vaccine-induced CD4 T cells across vaccine trails. By mapping phenotypic changes in these responses as a function of the antigen and pre-vaccination immune background, we aimed to address two questions: (i) does the polyfunctional phenotype of the vaccine-induced CD4 T cells depend on pre-existing immune memory; and (ii) does the vaccine-induced T cell response preferentially engage memory CD4 T cells, or does it also mobilize naïve CD4 T cells? Ultimately, an improved understanding the mechanisms underlying CD4 T-cell functionality can inform future vaccine design.

## Impact of host scenarios on CD4 T-cell phenotypes

Consistent with the early IFN-γ production in lymph nodes of AS01-injected animals (36), experimental AS01-adjuvanted vaccines were shown to elicit IFN-centered innate responses in naïve adults. When combined with HBs, two doses of AS01 induced robust innate transcriptional responses in whole blood, characterized by transient upregulation of several different modules comprising IFN-pathway and innate-cell-related (i.e., myeloid cell-related) genes, and downregulation of modules comprising lymphoid-cell-related signatures, including NK-cell and adaptive-cell-related genes (10, 11). These responses were accompanied by pro-inflammatory cytokine signals, notably IFN-γ and IP-10, detected in serum after the second dose. Note that these innate cytokine and transcriptional responses were predominantly detected after the second dose, while the responses detected after the first dose were much lower (10, 11). Based on data in mice, innate IFN-γ is likely produced by NK cells and/or antigen non-cognate CD8 T cells (36), in contrast to adaptive IFN-γ produced by CD4 T cells. Transient changes in responses of neutrophils, monocytes, dendritic cells and lymphocytes, as well as increased HLA-DR expression on monocytes, were also observed (10, 11). Collectively, these innate signals supported the activation of polyfunctional CD4 T cells, which in turn promoted CD4 T-cell differentiation into T_FH_ cells, a cell population that has been detected in human RTS,S/AS01 vaccinees (37). Efficacy of RZV/AS01, M72/AS01, RTS,S/AS01 and RSVPreF3/AS01 has been demonstrated in individuals with variable pre-exposure statuses (38–43). This raised the question whether CD4 T-cell phenotypes induced by AS01-adjuvanted licensed or candidate vaccines in individuals with distinct levels of antigen priming, differ from those induced in individuals with a naïve background. To address that question, we inspected the associated trial datasets for each of these vaccines (as summarized in **Table 1**).

### HBs/AS01

A Phase II study designed to facilitate longitudinal adjuvant comparisons included full-dose and half-dose AS01 along with three other adjuvants, each combined with HBs (8). Each vaccine was administered as a two-dose regimen to HBs-naïve young adults. Among the main expression markers (CD40L, IL-2, IFN-γ and TNF), the peripheral HBs-specific CD4 T-cell responses in both AS01 groups produced either CD40L alone (~one-third of the response) or co-produced CD40L and IL-2 with/without TNF (8). By contrast, IFN-γ^+^ cells were only detected at very low frequencies. HBs/AS01 vaccines have not been tested in HBs-primed adults.

### RZV/AS01

In RZV/AS01 efficacy trials, gE-specific CD4 T-cell responses were evaluated at 1, 12, 24 and 36 months post-dose 2 in VZV-exposed healthy older adults ≥50 or ≥70 years (41, 44). Responses were dominated by CD4 T cells expressing all four main expression markers, at proportions increasing over time in all age groups. It would be interesting to assess whether this increase in polyfunctionality is related to the persisting activation status of gE-specific CD4 T cells as recently reported (29). Among the main persisting CD4 T-cell populations, six different phenotypic profiles (in terms of the combinations of the four activation markers) were identified, two of which exhibiting IFN-γ expression (44) — in contrast with the IFN-γ responses to two HBs/AS01 doses in HBs-naïve individuals (8).

The vaccine has also been evaluated in IC populations including rheumatoid arthritis patients receiving Janus kinase (JAK) inhibitors, in whom induction of CD4 T-cell responses depended on JAK signaling (17). Interestingly, in RZV/AS01-vaccinated autologous HCT patients, the two-year persisting CD4 T-cell responses displayed a polyfunctionality profile highly similar to that in healthy older adults, with 2/5 main populations co-expressing IFN-γ alongside combinations of CD40L, IL-2, and TNF (18). Using IFN-γ, IL-2 and TNF markers, IFN-γ/IL-2–dominated CD4 T cell responses were observed in uremic immunodeficiency patients on hemodialysis (45). Moreover, lung transplant patients displayed similar populations but with more dominant IFN-γ–negative (particularly IL-2/CD40L-co-expressing) cells (19).

As most of the gE-specific CD4 T cells expanding post RZV/AS01 vaccination originate from memory cells (29), they would have been primed by childhood VZV infection(s). Hypothetically, this could be related to the greater capacity of the responding CD4 T cells to produce IFN-γ, as compared to the HBs-specific CD4 T cells not producing IFN-γ detected in naïve settings (8). This suggests that vaccine-expanded CD4 T cells originating from memory cells have acquired the capacity to produce IFN-γ, whilst CD4 T cells originating from naïve cells lack this capacity.

### M72/AS01

Mtb-specific Th1/Th17 CD4 T cells may be critical for protection against TB (46). A Phase 2b study of M72/AS01 showed that two vaccine doses conferred partial (49.7%) efficacy in latently TB-infected (LTBI) young adults in Year 3 (38). As part of the vaccine’s clinical development, populations with variable priming/memory backgrounds were evaluated, allowing assessment of different forms of priming: the same vaccine (or its comparable predecessor Mtb72F/AS02) was studied in infants, and in adults who were naïve, BCG-immunized/Mtb-unexposed, LTBI, previously TB-treated, or receiving TB treatment during the study.

Early Mtb72F/AS02 or Mtb72F/AS01 studies in naïve adults in non-endemic settings (47) revealed CD4 T-cell polyfunctionality profiles that were predominantly characterized by IL-2^+^ IFN-γ^−^ M72-specific CD4 T cells, as was confirmed in subsequent M72/AS01 studies (34, 48, 49).

A different polyfunctionality phenotype was observed in BCG-primed adults in settings with a distinct TB incidence. In Switzerland, four immune marker-expressing CD4 T-cell responses following Mtb72F/AS02 administration were detected in both BCG-vaccinated purified protein derivative-positive (PPD^+^) participants and previously Mtb-infected participants (50). Also in Switzerland, this phenotype was observed with M72/AS01 in people living with HIV/acquired immunodeficiency syndrome (AIDS) (PLWHA) on antiretroviral therapy (ART) (51), and dominated the response in BCG-primed PPD^+^ vaccinees in the Philippines (52). Similarly, polyfunctional IFN-γ−expressing cells represented a major fraction of M72-specific CD4 T cells in PLWHA and HIV-negative vaccinees in India who were mostly highly primed (PPD^+^: 67-77%; QuantiFERON TB test-positive (QFT^+^): 20-67%) (53), and this profile remained stable for ≤3 years post-vaccination (54). Of note, relative to Switzerland, the Philippines and India have a high TB incidence (3 versus 625 and 187 cases per 100,000, respectively, in 2024 (55)).

The dominance of IFN-γ^+^ CD4 T cells increased in M72/AS01 vaccinees living in high TB-burden South African areas (2024 TB incidence: 389/100,000 (55)). Two studies used the 9-color ICS assay, and study populations comprised BCG-vaccinated PPD-positive adults and adolescents, including LTBI individuals (33, 35). CD4 T-cell responses were dominated by IFN-γ−(co)-producing cells; no IL-17 was detected. As described for RTS,S/AS01 (56), a proportion of the IFN-γ was produced by NK cells that were activated by CD4 T cell-produced IL-2 (33). In an Mtb-primed, mixed IFN-γ release assay (IGRA)-positive/IGRA-negative cohort of PLWHA vaccinees in South Africa, the quadruple-positive CD4 T-cell subset was also detected, but single cytokine TNF-producing CD4 T cells dominated after immunization (57).

The impact of priming was further demonstrated by immune responses in adults who were either TB-naïve/BCG-primed, previously TB-treated, or had completed the intensive phase of TB treatment (24). In all three groups, IFN-γ−producing CD4 T cells dominated, and, as in the South African studies (33, 35), no IL-17- or IL-13-producing cells were detected (24).

Finally, responding CD4 T cells in Mtb-naïve M72/AS01-vaccinated infants displayed a naïve-like profile (58), with mainly CD40L^+^ IL-2^+^ cells, lower frequencies of CD40L^+^ IL-2^+^ TNF^+^ cells, and low proportions of quadruple-positive cells.

Collectively, the Mtb72F/AS01, Mtb72F/AS02 and M72/AS01 datasets suggest that the IFN-γ-production capacity of antigen-specific CD4 T cells increases with the level of immune priming, from the IL-2^+^ IFN-γ^−^ CD4 T cells mainly seen in naïve PPD-negative individuals, to the IFN-γ–producing CD4 T cells dominating the response in PPD-positive and LTBI participants in high-incidence settings.

### HIV-1 vaccines

While significant progress has been made in ART and prevention strategies, a safe, effective and accessible HIV vaccine remains elusive (59). AS01 was initially used to formulate the gp120-NefTat candidate, which was also evaluated with AS02 (60, 61), and later to formulate the F4-based candidate. The latter vaccine did not demonstrate viral efficacy in ART-naive, HIV-1-infected adults (62).

In HIV-1-seronegative adults, a 3- or 4-dose regimen of the gp120-NefTat/AS01 vaccine predominantly elicited CD4 T cells co-expressing TNF and IL-2, as well as CD40L (when included in the staining panels), followed by cells expressing TNF only, and then by cells expressing IL-2, either alone or with TNF and IFN-γ (14, 63). This profile was seen at both 3 months and 14 years post-vaccination, with the highest persistence observed for the quadruple-positive cells. Similarly, in naïve participants, 2 doses of the F4/AS01 vaccine induced mainly polyfunctional CD40L^+^subsets expressing IL-2 with or without TNF, while a small fraction of these cells also co-expressed IFN-γ (15, 64), mirroring observations in naïve HBs/AS01 and M72/AS01 vaccinees (8, 34, 48).

In HIV-1-infected ART-experienced volunteers, the gp120-NefTat/AS02 vaccine elicited CD40L^+/-^ CD4 T-cell responses producing IL-2 and/or IFN-γ at 6 months, whereas the response level of TNF^+^ cells was not statistically significant (61). For F4/AS01, the responding CD40L^+^ CD4 T cells consisted mostly of IL-2^+^ IFN-γ^+^ and IL-2^+^ TNF^+^ cells, followed by cells co-secreting IL-2, TNF and IFN-γ (65). These profiles were maintained up to Year 1 and persisted at lower frequencies in the ART-naïve compared with the ART-experienced individuals. Interestingly, the F4-specific triple cytokine-expressing phenotype was detected at higher frequencies in viral controllers than in other PLWHA categories, and showed the strongest correlations with viral load reduction following *in vitro* antigenic stimulation (66).

### RTS,S/AS01

The RTS,S/AS01 Phase 3 study in children revealed 46% efficacy during 18 months post-dose 3 (42), while a combination of RTS,S/AS01 (3 primary doses and 2 boosters) and chemoprevention conferred ≥60% efficacy within Year 1 (67). During the vaccine’s development, CSP-specific CD4 T-cell responses were assessed in whole blood from young children living in endemic regions, using an ICS assay measuring IL-2, IFN-γ and TNF expression (68). The data (excluded from **Table 1**) showed the strongest responses for IL-2^+^ CD4 T cells, followed by TNF^+^ cells, and only weak IFN-γ^+^ responses (68).

**Table 1:** CD4 T cell cytokine expression profiles induced by AS01-adjuvanted vaccines.

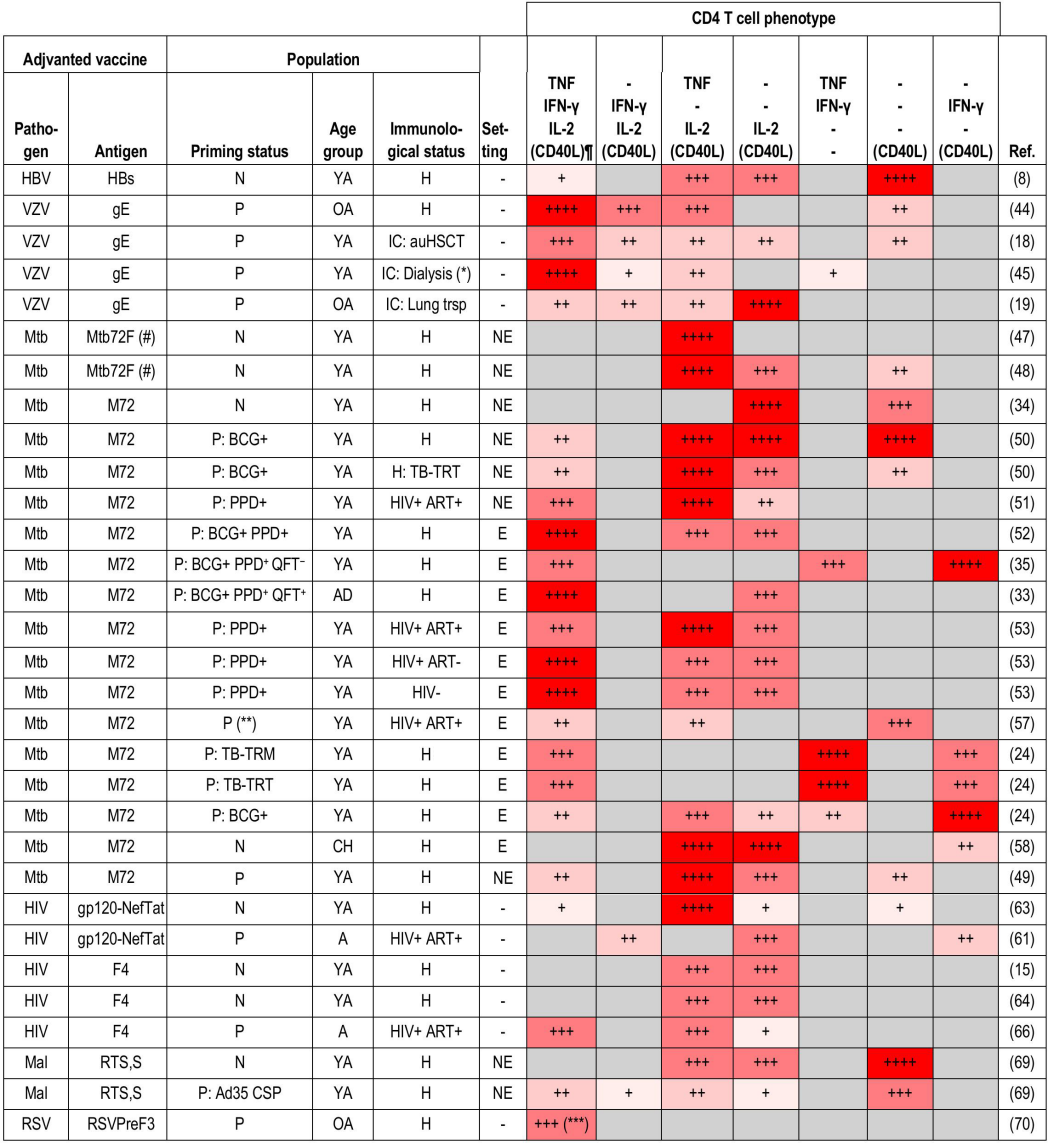

HBV, hepatitis B virus. VZV, varicella zoster virus. Mtb, *Mycobacterium tuberculosis*. HIV, human immunodeficiency virus. Mal, malaria. RSV, respiratory syncytial virus. HBs, hepatitis B surface antigen. gE, glycoprotein E. N/P, naïve/primed. BCG, Bacille Calmette-Guérin vaccine. PPD, purified protein derivative. QFT, QuantiFERON-TB test. TB-TRM, receiving tuberculosis treatment. TB-TRT, having received tuberculosis treatment. Ad35 CSP, adenovirus 35 vector circumsporozoite protein. A, adults, YA, young adults. OA, older adults. AD, adolescents. CH, young children. H, healthy. IC, immunocompromised. auHSCT, autologous hematopoietic stem cell transplant recipient. Lung trsp, lung transplant recipient. ART, antiretroviral therapy. -, not applicable. NE, non-endemic. E, endemic. (#) Mtb72F, predecessor antigen of M72, which was in this study adjuvanted with AS02, an oil-in-water emulsion containing MPL and QS21. (*), CD40L not included in intracellular cytokine staining assay. (**), data presented derived from a combined cohort of participants with a positive or negative interferon-g release assay status. Of note, the large proportions of TNF single-positive and TNF/CD40L-double-positive cells are not shown in the Table. (***), Only RSVPreF3-specific CD4 T cells expressing IFN-γ or at least 2 markers amongst IL-2, IFN-γ, TNF and CD40L were reported, limiting determination of the detailed polyfunctionality profile. Refs (31, 49, 68) are not included in this table due to different data presentations or lack of reported CD4 T cell responses. (¶) (CD40L) indicates that some (but not all) studies included the CD40L marker.

A similar phenotype was observed in malaria-naïve adults in the US (69). A three-dose RTS,S/AS01 regimen was compared to a regimen in which a priming dose of CSP-expressing Adenovirus 35 vector (Ad35) was followed by two RTS,S/AS01 doses, allowing comparison of the effects of Ad35 vs RTS,S/AS01 priming. Consistent with earlier data in naïve individuals (8, 34, 48), the predominant phenotype of CSP-specific CD4 T-cell responses in the RTS,S-only study arm were IL-2^+^ TNF^+/-^ CD40L^+^ cells with low IFN-γ expression. In contrast, Ad35-priming followed by two RTS,S/AS01 doses resulted in a far stronger IFN-γ response in the polyfunctional CSP-specific CD4 T cells, again highlighting the role of immunological priming in driving CD4 T-cell phenotypes.

### RSVPreF3/AS01

RSV (re)infections occur frequently from an early age onwards (potentially causing complications in IC and/or older individuals), and RSV-specific CD4 T-cell responses offer a prime example of often reactivated CD4 T-cell memory. An RSVPreF3/AS01 Phase1/2 study (70) in immune-primed older adults who received two doses showed similar frequencies of RSVPreF3-specific CD4 T cells for phenotypes expressing at least IFN-γ (among IFN-γ, IL-13, and IL-17) and phenotypes expressing ≥2 markers (70), indicating that most of the vaccine-induced CD4 T cells secreted IFN-γ. A comparable profile was observed in volunteers receiving non-adjuvanted RSVPreF3 vaccine, indicating that the adjuvant did not drive this phenotype (70).

Altogether, in a naïve background, responding CD4 T-cell populations are dominated by IL-2^+^ TNF^+^/^-^IFN-γ^−^ cells, as observed in trials in naive adults receiving HBs/AS01, F4/AS01, gp120-NefTat/AS01 and M72/AS01, and in (young) children in endemic regions receiving M72/AS01 or RTS,S/AS01. Thus, in the presence of immune memory—e.g. older adults receiving RZV/AS01, or PPD^+^ QFT^+^ M72/AS01 vaccinees—proportions of IFN-γ^+^ cells increase and start to dominate the CD4 T-cell response. The RSVPreF3/AS01 data in older adults further indicate that IFN-γ^+^ phenotypes dominate in the presence of immune memory. AS01 enhances the overall response magnitude but did not appear to change the CD4 T-cell phenotypes seen in this study (70).

## Discussion

The precise cellular origin, i.e. memory versus naïve, of CD4 T cells responding to AS01-adjuvanted vaccines remains unclear. To determine how both the vaccine antigen and the host’s pre-vaccination immune status shape phenotypic changes in these cells, we analyzed vaccine-induced CD4 T-cell responses observed in clinical trials evaluating AS01-adjuvanted vaccines.

**Figure 1:**
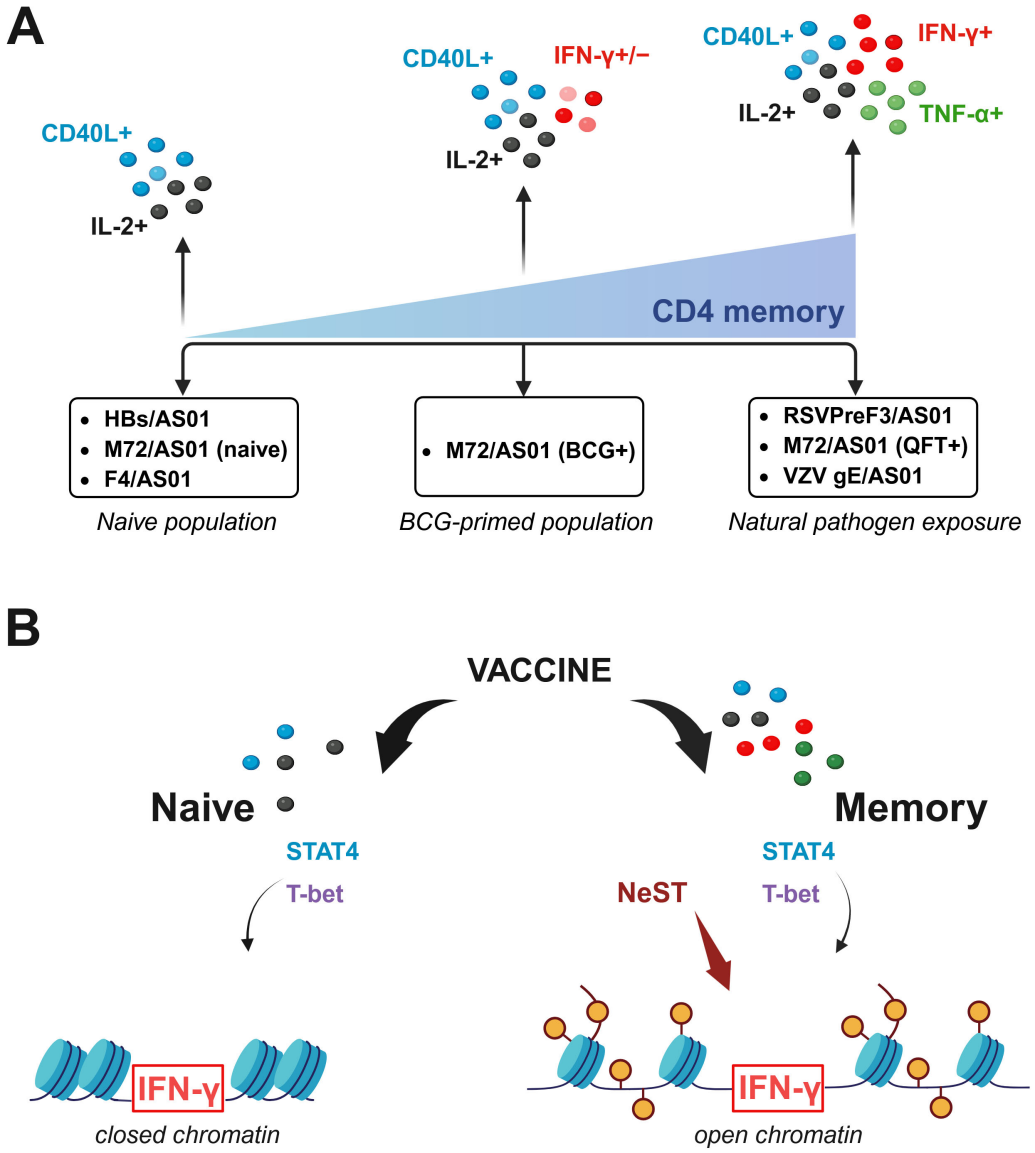
CD4 T cell memory affects CD4 T cell cytokine expression profiles. **(A)** Illustration of the relationship between pre-existing CD4 T cell memory and polyfunctionality. In the absence of pre-existing memory, CD4 T cells express IL-2 and some TNF but not IFN-γ, exemplified by responses to the hepatitis B virus surface antigen (HBs)/AS01, the human immunodeficiency virus type 1 (HIV-1) vaccines F4/AS01 and gp120-NefTat in naïve populations, as well as the M72/AS01 tuberculosis (TB) vaccine in a TB/Bacille Calmette-Guérin (BCG)-naïve background of the host. IFN-γ-producing CD4 T cells become dominant with increasing levels of prior exposure leading to pre-existing memory, exemplified by the responses to the recombinant varicella zoster virus glycoprotein E (VZV gE)/AS01 vaccine and the respiratory syncytial virus (RSV) vaccine RSVPreF3/AS01 vaccine in primed individuals, to either of the HIV vaccines in primed populations on antiretroviral (ART) therapy, and to the M72/AS01 vaccine in primed adults in pathogen-endemic areas. **(B)** A proposed mechanism how AS01-adjuvanted vaccines can drive different polyfunctionality profiles based on different immune backgrounds. Whereas STAT4 signaling is expected to be part of the AS01 mode of action, epigenetic control at the chromatin level could lead to different IFN-γ expression levels. The IFN locus is regulated by the long-noncoding RNA Nettoie Salmonella pas Theiler's (NeST), that could be differentially activated in memory versus naive CD4 T cells. Created with BioRender.com.

Comparison of distinct immune backgrounds revealed a consistent difference in CD4 T-cell polyfunctionality profiles. Across all vaccines examined, the IFN-γ-production capacity of antigen-specific, vaccine-induced CD4 T cells appeared to depend on the presence and magnitude of pre-existing memory (**Figure 1A**). M72/AS01 development studies were particularly informative, as they enabled evaluating CD4 T-cell responses across varying levels and types of pre-existing CD4 T-cell memory. BCG- and mTB-naïve participants predominantly displayed IL-2^+^ TNF^+/-^ IFN-γ^−^ CD4 T-cell responses, while responses in BCG-immunized vaccinees comprised both IFN-γ^+^ and IFN-γ^−^ CD4 T cells, and a QFT^+^ status was associated with a dominance of IFN-γ^+^ CD4^+^ T cells. Similarly, immunization with RSV or VZV gE antigens, for which most individuals possess pre-existing CD4 T-cell memory, resulted in the preferential expansion of IFN-γ^+^ CD4 cells (18, 44, 70).

The collective data suggest that AS01-adjuvanted vaccines generate IL-2^+^ CD4 T cells, but, in the absence of immune memory, do not efficiently elicit IFN-γ expression by CD4 T cells. In contrast, when CD4 T-cell memory has been established through prior infection (e.g. by VZV, Mtb, RSV, or HIV) or immunization (e.g., by BCG), IFN-γ^+^ CD4 T cells expand and can dominate the vaccine-induced response. Although the association between pre-existing immune memory and IFN-γ production was consistent across these datasets, definitive evidence, including TCR repertoire analyses, is required to substantiate this interpretation. Currently, the scRNA-seq dataset reported for RZV/AS01 (29) provides the most informative resource to address this. Notably, the authors’ conclusion that the majority of vaccine-expanded CD4 T cells originate from pre-existing memory populations aligns with the predominance of IFN-γ^+^ memory-derived CD4 T-cell phenotype observed in the trials in older and IC adults (18, 44). Extending such scRNA-seq CD4 T-cell analyses to additional AS01-adjuvanted vaccines, particularly M72/AS01 and RTS,S/AS01, in endemic settings can allow linking CD4 T-cell phenotypes to clinical outcomes and would thus be highly informative for next-generation vaccine development. Moreover, it is important to determine whether this paradigm extends to other adjuvants. Indeed, the AS03 emulsion-based adjuvant appears to follow a similar pattern: the investigational HBs/AS03 vaccine induced IL-2^+^ IFN-γ^-^ CD4 T cells in naïve participants (8), whereas AS03-adjuvanted seasonal influenza vaccines administered in previously exposed populations elicited IFN-γ^+^ CD4 T-cell responses (71).

Single-cell transcriptomic and TCR-repertoire analyses are required to test whether the IFN-γ production capacity would be unlocked only by prior infection or immunization. With this caveat in mind, the mechanistic basis of this phenomenon warrants closer examination. As CD4 T-cell and NK-cell IFN-γ production depends on IL-12-mediated signal transducers and activators of transcription (STAT)4 signaling (72), one explanation for the lack of IFN-γ production in CD4 T cells in naïve vaccinees is that AS01 may not induce a sufficiently robust STAT4-activating signal. However, several lines of evidence argue against a deficient IL-12/STAT4 axis as primary explanation:

– AS01-adjuvanted vaccines induce IL-12 (36).
– IFN-γ is detected in human serum one day post-vaccination with HBs/AS01 (10) or M72/AS01 (49) in naïve individuals (in whom vaccine-induced CD4 T cells do not secrete IFN-γ), indicating that the capacity to produce IFN-γ is intact.
– Transcriptomics analyses reveal a transient IFN-response signature emerging post-vaccination with HBs/AS01 (11), M72/AS01 (49) or RTS,S/AS01 (69) in immunologically naïve individuals.
– CD4 T-cell responses to RZV/AS01 are strongly reduced in the presence of JAK inhibitors (17), suggesting that JAK/STAT signaling is required and unlikely to be the limiting factor governing IFN-γ production.
– Antigen-dependent NK-cell IFN-γ production in African children immunized with RTS,S/AS01 is IL-2-dependent, driven by CD4 T cell-derived IL-2 (56). A similar observation was made for M72/AS01 in BCG-vaccinated adolescents living in South Africa (33).

Collectively, this indicates that STAT4 signaling is intact following AS01-adjuvanted vaccination. RZV/AS01 and M72/AS01 studies in BCG-immunized or Mtb-infected participants further demonstrate that vaccine-induced CD4 T cells do not exhibit an intrinsic defect in activating the pathways required for IFN-γ production. Alternatively, IFN-γ expression could be regulated at the epigenetic level, such that differences between naive and primed CD4 T cells reflect differential accessibility of the IFN-γ locus (**Figure 1B**). This would be consistent with the conclusion that long-term transcriptional changes in RZV-induced CD4 T cells are epigenetically programmed (29). Accessibility of the IFN-γ locus is epigenetically regulated by the long-noncoding RNA Nettoie Salmonella pas Theiler’s (NeST), which is activated by viral infection (73). Thus, the signal to activate NeST following viral, and potentially, mycobacterial infection, may be absent following AS01-adjuvanted vaccination. Consequently, only when the IFN-γ locus is permissive at the chromatin level are IFN-γ^+^ CD4 T cells preferentially expanded. Generating scATAC-seq data to map chromatin accessibility at the different cytokine loci in CD4 T cells, is required to directly test this hypothesis.

## Perspective

IFN-γ expression in CD4 T cells after vaccination appears to be tightly controlled. The implications of the differential CD4 T-cell polyfunctionality patterns on antibody or CD8 T-cell responses are not so clear, however. The AS01 adjuvant itself has a strong impact on antibody functionality. Compared to HBs/alum, AS01-adjuvanted HBs vaccines induced high-avidity antibody responses, including total Ig, IgG_1_, IgA_1_, IgM, and Fc-dependent antibody responses such as antibody dependent cellular cytotoxicity (ADCC) (12, 74, 75). These antibody functionalities correlated with an innate IFN signature (12, 74) but were underpinned by IL-2^+^ IFN-γ-negative CD4 T cells, since this was the dominant CD4 phenotype in that study (8). Unfortunately, there are currently no comparative data that address the effect of IFN-γ expression in CD4 T cells on antibody properties or functionality. Single-cell data would be key to address this issue in future studies. While CD8 T-cell responses can also be critical for protection, they have not been detected after vaccination with HBs/AS01 in an immunologically naïve background (8). In contrast, M72/AS01 induced transient CD8 T-cell responses at 7 days post vaccination in QFT^+^ but not QFT^−^ individuals (33). However, CD4 T-cell *frequencies* also correlated with QFT status (33), thereby confounding the interpretation of the potential effects of CD4 T-cell polyfunctionality. Therefore, investigating the downstream impact of CD4 T-cell polyfunctionality on effector mechanisms remains an important avenue for research. Despite uncertainty regarding the functional implications, understanding the regulation of IFN-γ expression is important for the rational design of novel adjuvants. Adjuvants that more closely mimic viral infection and activate NeST-dependent pathways may induce distinct polyfunctionality profiles. Interestingly, early studies of SARS-CoV-2 spike mRNA-lipid nanoparticle vaccines in immunologically naïve individuals reported the induction of polyfunctional IFN-γ^+^ CD4 T cells (76, 77). An important question is by which specific mechanisms different immune backgrounds in target populations, such as older adults, IC individuals, or individuals with different exposure histories, influence the quality and polyfunctionality of vaccine-induced T-cell responses. This remains an area requiring further research.

A strength of our assessment is that most of the CD4 T-cell data were generated using the same validated assay, enhancing inter-study comparability. Limitations included (i) the relatively small number of available datasets and limited analysis of additional cytokines; (ii) the exclusive reliance on blood- or PBMC-derived CD4 T-cell data, lacking lymph node analyses; (iii) the incomplete data stratification by population priming status (e.g. mixed IGRA-positive/negative populations (57)); (iv) the relative paucity of TCR-repertoire data; and (v), the lack of epigenomics profiling data.

Altogether, analysis of the collective post-vaccination CD4 T-cell data reveals a consistent pattern in which IFN-γ expression is associated with the presence of pre-existing memory T cells. Conversely, this observation suggests that IFN-γ^+^ CD4 T cells induced by the reviewed AS01-adjuvanted vaccines predominantly originate from memory cells. This analysis can guide further research into improving the effectiveness of next-generation vaccines targeting specific populations.

## Data Availability

The original contributions presented in the study are included in the article/supplementary material. Further inquiries can be directed to the corresponding author.

## References

[1] Plotkin SA . Correlates of protection induced by vaccination. Clin Vaccine Immunol. (2010) 17:1055–65. doi: 10.1128/cvi.00131-10 20463105 PMC2897268

[2] Crotty S . T follicular helper cell biology: A decade of discovery and diseases. Immunity. (2019) 50:1132–48. doi: 10.1016/j.immuni.2019.04.011 31117010 PMC6532429

[3] Lewinsohn DA Lewinsohn DM Scriba TJ . Polyfunctional CD4^+^ T cells as targets for tuberculosis vaccination. Front Immunol. (2017) 8:1262. doi: 10.3389/fimmu.2017.01262 29051764 PMC5633696

[4] Cunningham AL Sandgren KJ Truong NR . Advances in understanding the mechanism of action of adult vaccines. J Clin Invest. (2023) 133(23):e175378. doi: 10.1172/jci175378 38038131 PMC10688986

[5] Boyd A Almeida JR Darrah PA Sauce D Seder RA Appay V . Pathogen-specific T cell polyfunctionality is a correlate of T cell efficacy and immune protection. PLoS One. (2015) 10:e0128714. doi: 10.1371/journal.pone.0128714 26046523 PMC4457486

[6] Seder RA Darrah PA Roederer M . T-cell quality in memory and protection: Implications for vaccine design. Nat Rev Immunol. (2008) 8:247–58. doi: 10.1038/nri2274 18323851

[7] Scriba TJ Netea MG Ginsberg AM . Key recent advances in TB vaccine development and understanding of protective immune responses against Mycobacterium tuberculosis. Semin Immunol. (2020) 50:101431. doi: 10.1016/j.smim.2020.101431 33279383 PMC7786643

[8] Leroux-Roels G Marchant A Levy J Van Damme P Schwarz TF Horsmans Y . Impact of adjuvants on CD4^+^ T cell and B cell responses to a protein antigen vaccine: Results from a phase II, randomized, multicenter trial. Clin Immunol. (2016) 169:16–27. doi: 10.1016/j.clim.2016.05.007 27236001

[9] Roman F Burny W Ceregido MA Laupèze B Temmerman ST Warter L . Adjuvant system AS01: From mode of action to effective vaccines. Expert Rev Vaccines. (2024) 23:715–29. doi: 10.1080/14760584.2024.2382725 39042099

[10] Burny W Callegaro A Bechtold V Clement F Delhaye S Fissette L . Different adjuvants induce common innate pathways that are associated with enhanced adaptive responses against a model antigen in humans. Front Immunol. (2017) 8:943. doi: 10.3389/fimmu.2017.00943 28855902 PMC5557780

[11] De Mot L Bechtold V Bol V Callegaro A Coccia M Essaghir A . Transcriptional profiles of adjuvanted hepatitis B vaccines display variable interindividual homogeneity but a shared core signature. Sci Transl Med. (2020) 12(569):eaay8618. doi: 10.1126/scitranslmed.aay8618 33177181

[12] Tasdighian S Bechtold V Essaghir A Saeys Y Burny W . An innate immune signature induced by AS01- or AS03-adjuvanted vaccines predicts the antibody response magnitude and quality consistently over time. Front Immunol. (2024) 15:1412732. doi: 10.3389/fimmu.2024.1412732 39206189 PMC11349632

[13] Bechtold V Smolen KK Burny W de Angelis SP Delandre S Essaghir A . Functional and epigenetic changes in monocytes from adults immunized with an AS01-adjuvanted vaccine. Sci Transl Med. (2024) 16:eadl3381. doi: 10.1126/scitranslmed.adl3381 39083587

[14] Leroux-Roels I Koutsoukos M Clement F Steyaert S Janssens M Bourguignon P . Strong and persistent CD4^+^ T-cell response in healthy adults immunized with a candidate HIV-1 vaccine containing gp120, Nef and Tat antigens formulated in three adjuvant systems. Vaccine. (2010) 28:7016–24. doi: 10.1016/j.vaccine.2010.08.035 20728522

[15] Van Braeckel E Bourguignon P Koutsoukos M Clement F Janssens M Carletti I . An adjuvanted polyprotein HIV-1 vaccine induces polyfunctional cross-reactive CD4^+^ T cell responses in seronegative volunteers. Clin Infect Dis. (2011) 52:522–31. doi: 10.1093/cid/ciq160 21208909 PMC3060898

[16] Lötscher J Walti CS Heller S Hengy Linder F Drexler B Gerull S . Respiratory syncytial virus vaccination in adult allogeneic hematopoietic cell transplant recipients. JAMA. (2025) 334:1478–80. doi: 10.1001/jama.2025.16744 PMC12492292 41037295

[17] Kallmark H Gullstrand B Kahn F Grenmyr E Kahn R Einarsson JT . Impaired cellular immune responses to herpes zoster subunit vaccine in patients with rheumatoid arthritis receiving Janus kinase inhibitors. Rheumatol. (2025) 65(1):keaf626. doi: 10.1093/rheumatology/keaf626 PMC12861083 41330694

[18] Stadtmauer EA Sullivan KM El Idrissi M Salaun B Alonso Alonso A Andreadis C . Adjuvanted recombinant zoster vaccine in adult autologous stem cell transplant recipients: Polyfunctional immune responses and lessons for clinical practice. Hum Vaccin Immunother. (2021) 17:4144–54. doi: 10.1080/21645515.2021.1953346 34406911 PMC8828160

[19] Hirzel C L’Huillier AG Ferreira VH Marinelli T Ku T Ierullo M . Safety and immunogenicity of adjuvanted recombinant subunit herpes zoster vaccine in lung transplant recipients. Am J Transplant. (2021) 21:2246–53. doi: 10.1111/ajt.16534 33565711 PMC9169546

[20] Sadowska-Klasa A Lim FY Xie H Zamora D Stevens-Ayers T Leisenring WM . New insights into factors shaping CMV-specific T-cell polyfunctionality after hematopoietic cell transplantation. Am J Hematol. (2026) 101:255–68. doi: 10.1002/ajh.70152 41324241 PMC12766359

[21] Gustafson CE Kim C Weyand CM Goronzy JJ . Influence of immune aging on vaccine responses. J Allergy Clin Immunol. (2020) 145:1309–21. doi: 10.1016/j.jaci.2020.03.017 32386655 PMC7198995

[22] Dintwe OB Day CL Smit E Nemes E Gray C Tameris M . Heterologous vaccination against human tuberculosis modulates antigen-specific CD4^+^ T-cell function. Eur J Immunol. (2013) 43:2409–20. doi: 10.1002/eji.201343454 PMC3816254 23737382

[23] Coccia M Burny W Demoitié MA Gillard P van den Berg RA van der Most R . Subsequent AS01-adjuvanted vaccinations induce similar transcriptional responses in populations with different disease statuses. PLoS One. (2022) 17:e0276505. doi: 10.1371/journal.pone.0276505 36355775 PMC9648731

[24] Gillard P Yang PC Danilovits M Su WJ Cheng SL Pehme L . Safety and immunogenicity of the M72/AS01_E_ candidate tuberculosis vaccine in adults with tuberculosis: A phase II randomised study. Tuberculosis (Edinb). (2016) 100:118–27. doi: 10.1016/j.tube.2016.07.005 27553419

[25] Zimmermann P Curtis N . Factors that influence the immune response to vaccination. Clin Microbiol Rev. (2019) 32(2):e00084-18. doi: 10.1128/cmr.00084-18 30867162 PMC6431125

[26] Purcell RA Theisen RM Arnold KB Chung AW Selva KJ . Polyfunctional antibodies: A path towards precision vaccines for vulnerable populations. Front Immunol. (2023) 14:1183727. doi: 10.3389/fimmu.2023.1183727 37600816 PMC10433199

[27] Lynn DJ Benson SC Lynn MA Pulendran B . Modulation of immune responses to vaccination by the microbiota: Implications and potential mechanisms. Nat Rev Immunol. (2022) 22:33–46. doi: 10.1038/s41577-021-00554-7 34002068 PMC8127454

[28] Scanlon N Saklawi Y Rouphael N . The role of systems vaccinology in understanding the immune defects to vaccination in solid organ transplant recipients. Front Immunol. (2020) 11:582201. doi: 10.3389/fimmu.2020.582201 33324400 PMC7723964

[29] Wen X Hu AK Presnell SR Ford ES Koelle DM Kwok WW . Longitudinal single cell profiling of epitope specific memory CD4^+^ T cell responses to recombinant zoster vaccine. Nat Commun. (2025) 16:2332. doi: 10.1038/s41467-025-57562-7 40057520 PMC11890790

[30] Laing KJ Ford ES Johnson MJ Levin MJ Koelle DM Weinberg A . Recruitment of naive CD4^+^ T cells by the recombinant zoster vaccine correlates with persistent immunity. J Clin Invest. (2023) 133(23):e172634. doi: 10.1172/jci172634 37788096 PMC10688978

[31] Moris P Bellanger A Ofori-Anyinam O Jongert E Yarzabal Rodriguez JP Janssens M . Whole blood can be used as an alternative to isolated peripheral blood mononuclear cells to measure *in vitro* specific T-cell responses in human samples. J Immunol Methods. (2021) 492:112940. doi: 10.1016/j.jim.2020.112940 33493551

[32] Moris P Jongert E van der Most RG . Characterization of T-cell immune responses in clinical trials of the candidate RTS,S malaria vaccine. Hum Vaccin Immunother. (2018) 14:17–27. doi: 10.1080/21645515.2017.1381809 28934066 PMC5791571

[33] Penn-Nicholson A Geldenhuys H Burny W van der Most R Day CL Jongert E . Safety and immunogenicity of candidate vaccine M72/AS01_E_ in adolescents in a TB endemic setting. Vaccine. (2015) 33:4025–34. doi: 10.1016/j.vaccine.2015.05.088 26072017 PMC5845829

[34] Leroux-Roels I Forgus S De Boever F Clement F Demoitié MA Mettens P . Improved CD4^+^ T cell responses to Mycobacterium tuberculosis in PPD-negative adults by M72/AS01 as compared to the M72/AS02 and Mtb72F/AS02 tuberculosis candidate vaccine formulations: A randomized trial. Vaccine. (2013) 31:2196–206. doi: 10.1016/j.vaccine.2012.05.035 22643213

[35] Day CL Tameris M Mansoor N van Rooyen M de Kock M Geldenhuys H . Induction and regulation of T-cell immunity by the novel tuberculosis vaccine M72/AS01 in South African adults. Am J Respir Crit Care Med. (2013) 188:492–502. doi: 10.1164/rccm.201208-1385oc 23306546 PMC3778736

[36] Coccia M Collignon C Hervé C Chalon A Welsby I Detienne S . Cellular and molecular synergy in AS01-adjuvanted vaccines results in an early IFNγ response promoting vaccine immunogenicity. NPJ Vaccines. (2017) 2:25. doi: 10.1038/s41541-017-0027-3 29263880 PMC5627273

[37] Pallikkuth S Chaudhury S Lu P Pan L Jongert E Wille-Reece U . A delayed fractionated dose RTS,S AS01 vaccine regimen mediates protection via improved T follicular helper and B cell responses. Elife. (2020) 9:e51889. doi: 10.7554/elife.51889 32342859 PMC7213985

[38] Tait DR Hatherill M Van Der Meeren O Ginsberg AM Van Brakel E Salaun B . Final analysis of a trial of M72/AS01_E_ vaccine to prevent tuberculosis. N Engl J Med. (2019) 381:2429–39. doi: 10.1056/nejmoa1909953 31661198

[39] Van Der Meeren O Hatherill M Nduba V Wilkinson RJ Muyoyeta M Van Brakel E . Phase 2b controlled trial of M72/AS01_E_ vaccine to prevent tuberculosis. N Engl J Med. (2018) 379:1621–34. doi: 10.1056/nejmoa1803484 30280651 PMC6151253

[40] Cunningham AL Lal H Kovac M Chlibek R Hwang SJ Diez-Domingo J . Efficacy of the herpes zoster subunit vaccine in adults 70 years of age or older. N Engl J Med. (2016) 375:1019–32. doi: 10.1056/nejmoa1603800 27626517

[41] Lal H Cunningham AL Godeaux O Chlibek R Diez-Domingo J Hwang SJ . Efficacy of an adjuvanted herpes zoster subunit vaccine in older adults. N Engl J Med. (2015) 372:2087–96. doi: 10.1056/nejmoa1501184 25916341

[42] RTS SCTP . Efficacy and safety of RTS,S/AS01 malaria vaccine with or without a booster dose in infants and children in Africa: Final results of a phase 3, individually randomised, controlled trial. Lancet. (2015) 386:31–45. doi: 10.1056/nejmoa1208394 25913272 PMC5626001

[43] Ison MG Papi A Athan E Feldman RG Langley JM Lee DG . Efficacy and safety of respiratory syncytial virus (RSV) prefusion F protein vaccine (RSVPreF3 OA) in older adults over 2 RSV seasons. Clin Infect Dis. (2024) 78:1732–44. doi: 10.1093/cid/ciae010 38253338 PMC11175669

[44] Cunningham AL Heineman TC Lal H Godeaux O Chlibek R Hwang SJ . Immune responses to a recombinant glycoprotein E herpes zoster vaccine in adults aged 50 years or older. J Infect Dis. (2018) 217:1750–60. doi: 10.1093/infdis/jiy095 29529222 PMC5946839

[45] Hielscher F Schmidt T Enders M Leyking S Gerhart M van Bentum K . The inactivated herpes zoster vaccine HZ/su induces a varicella zoster virus specific cellular and humoral immune response in patients on dialysis. EBioMedicine. (2024) 108:105335. doi: 10.1016/j.ebiom.2024.105335 39265505 PMC11416227

[46] Wang J Fan XY Hu Z . Immune correlates of protection as a game changer in tuberculosis vaccine development. NPJ Vaccines. (2024) 9:208. doi: 10.1038/s41541-024-01004-w 39478007 PMC11526030

[47] Von Eschen K Morrison R Braun M Ofori-Anyinam O De Kock E Pavithran P . The candidate tuberculosis vaccine Mtb72F/AS02_A_: Tolerability and immunogenicity in humans. Hum Vaccin. (2009) 5:475–82. doi: 10.4161/hv.8570 19587528

[48] Leroux-Roels I Leroux-Roels G Ofori-Anyinam O Moris P De Kock E Clement F . Evaluation of the safety and immunogenicity of two antigen concentrations of the Mtb72F/AS02_A_ candidate tuberculosis vaccine in purified protein derivative-negative adults. Clin Vaccine Immunol. (2010) 17:1763–71. doi: 10.1128/cvi.00133-10 20861328 PMC2976103

[49] van den Berg RA De Mot L Leroux-Roels G Bechtold V Clement F Coccia M . Adjuvant-associated peripheral blood mRNA profiles and kinetics induced by the adjuvanted recombinant protein candidate tuberculosis vaccine M72/AS01 in Bacillus Calmette-Guerin-vaccinated adults. Front Immunol. (2018) 9:564. doi: 10.3389/fimmu.2018.00564 29632533 PMC5879450

[50] Spertini F Audran R Lurati F Ofori-Anyinam O Zysset F Vandepapelière P . The candidate tuberculosis vaccine Mtb72F/AS02 in PPD positive adults: A randomized controlled phase I/II study. Tuberculosis (Edinb). (2013) 93:179–88. doi: 10.1016/j.tube.2012.10.011 23219236

[51] Thacher EG Cavassini M Audran R Thierry AC Bollaerts A Cohen J . Safety and immunogenicity of the M72/AS01 candidate tuberculosis vaccine in HIV-infected adults on combination antiretroviral therapy: a phase I/II, randomized trial. AIDS. (2014) 28:1769–81. doi: 10.1097/qad.0000000000000343 24911353

[52] Montoya J Solon JA Cunanan SR Acosta L Bollaerts A Moris P . A randomized, controlled dose-finding phase II study of the M72/AS01 candidate tuberculosis vaccine in healthy PPD-positive adults. J ClinImmunol. (2013) 33:1360–75. doi: 10.1007/s10875-013-9949-3 24142232 PMC3825318

[53] Kumarasamy N Poongulali S Bollaerts A Moris P Beulah FE Ayuk LN . A randomized, controlled safety, and immunogenicity trial of the M72/AS01 candidate tuberculosis vaccine in HIV-positive Indian adults. Med (Baltimore). (2016) 95:e2459. doi: 10.1097/md.0000000000002459 26817879 PMC4998253

[54] Kumarasamy N Poongulali S Beulah FE Akite EJ Ayuk LN Bollaerts A . Long-term safety and immunogenicity of the M72/AS01_E_ candidate tuberculosis vaccine in HIV-positive and -negative Indian adults: Results from a phase II randomized controlled trial. Med (Baltimore). (2018) 97:e13120. doi: 10.1097/md.0000000000013120 30407329 PMC6250513

[55] World Bank Group . Incidence of tuberculosis (per 100,000 people). Available online at: https://data.worldbank.org/indicator/SH.TBS.INCD?end=2024&start=2000&view=chart (Accessed May 25, 2026).

[56] Horowitz A Hafalla JC King E Lusingu J Dekker D Leach A . Antigen-specific IL-2 secretion correlates with NK cell responses after immunization of Tanzanian children with the RTS,S/AS01 malaria vaccine. J Immunol. (2012) 188:5054–62. doi: 10.4049/jimmunol.1102710 22504653 PMC3378032

[57] Dagnew AF Han LL Naidoo K Fairlie L Innes JC Middelkoop K . Safety and immunogenicity of investigational tuberculosis vaccine M72/AS01_E-4_ in people living with HIV in South Africa: an observer-blinded, randomised, controlled, phase 2 trial. Lancet HIV. (2025) 12:e546–55. doi: 10.1016/s2352-3018(25)00124-9 40614747 PMC12310912

[58] Idoko OT Owolabi OA Owiafe PK Moris P Odutola A Bollaerts A . Safety and immunogenicity of the M72/AS01 candidate tuberculosis vaccine when given as a booster to BCG in Gambian infants: an open-label randomized controlled trial. Tuberculosis (Edinb). (2014) 94:564–78. doi: 10.1016/j.tube.2014.07.001 25305000

[59] Shim I Rogowski L Venketaraman V . Progress and recent developments in HIV vaccine research. Vaccines. (2025) 13:690. doi: 10.3390/vaccines13070690 40733667 PMC12298520

[60] Goepfert PA Tomaras GD Horton H Montefiori D Ferrari G Deers M . Durable HIV-1 antibody and T-cell responses elicited by an adjuvanted multi-protein recombinant vaccine in uninfected human volunteers. Vaccine. (2007) 25:510–8. doi: 10.1016/j.vaccine.2006.07.050 17049679

[61] Lichterfeld M Gandhi RT Simmons RP Flynn T Sbrolla A Yu XG . Induction of strong HIV-1–specific CD4^+^ T-cell responses using an HIV-1 gp120/NefTat vaccine adjuvanted with AS02_A_ in antiretroviral-treated HIV-1–infected individuals. JAIDS J Acquired Immune Deficiency Syndromes. (2012) 59:1–9. doi: 10.1097/QAI.0b013e3182373b77 PMC3241906 21963936

[62] Dinges W Girard P-M Podzamczer D Brockmeyer NH García F Harrer T . The F4/AS01_B_ HIV-1 vaccine candidate is safe and immunogenic, but does not show viral efficacy in antiretroviral therapy-naive, HIV-1-infected adults: a randomized controlled trial. Medicine. (2016) 95:e2673. doi: 10.1016/j.vaccine.2018.03.043 26871794 PMC4753889

[63] Van Der Meeren O Jongert E Seaton KE Koutsoukos M Aerssens A Brackett C . Persistence of vaccine-elicited immune response up to 14 years post-HIV gp120-NefTat/AS01_B_ vaccination. Vaccine. (2020) 38:1678–89. doi: 10.1016/j.vaccine.2019.12.058 31932137

[64] Leroux-Roels G Bourguignon P Willekens J Janssens M Clement F Didierlaurent AM . Immunogenicity and safety of a booster dose of an investigational adjuvanted polyprotein HIV-1 vaccine in healthy adults and effect of administration of chloroquine. Clin Vaccine Immunol. (2014) 21:302–11. doi: 10.1128/cvi.00617-13 24391139 PMC3957681

[65] Harrer T Plettenberg A Arastéh K Van Lunzen J Fätkenheuer G Jaeger H . Safety and immunogenicity of an adjuvanted protein therapeutic HIV-1 vaccine in subjects with HIV-1 infection: a randomised placebo-controlled study. Vaccine. (2014) 32:2657–65. doi: 10.1016/j.vaccine.2013.10.030 24144472

[66] Van Braeckel E Desombere I Clement F Vandekerckhove L Verhofstede C Vogelaers D . Polyfunctional CD4^+^ T cell responses in HIV-1-infected viral controllers compared with those in healthy recipients of an adjuvanted polyprotein HIV-1 vaccine. Vaccine. (2013) 31:3739–46. doi: 10.1007/s10875-010-9490-6 23707169

[67] Cairns M Barry A Zongo I Sagara I Yerbanga SR Diarra M . The duration of protection against clinical malaria provided by the combination of seasonal RTS,S/AS01_E_ vaccination and seasonal malaria chemoprevention versus either intervention given alone. BMC Med. (2022) 20:352. doi: 10.1186/s12916-022-02536-5 36203149 PMC9540742

[68] Olotu A Moris P Mwacharo J Vekemans J Kimani D Janssens M . Circumsporozoite-specific T cell responses in children vaccinated with RTS,S/AS01_E_ and protection against P falciparum clinical malaria. PLoS One. (2011) 6:e25786. doi: 10.1371/journal.pone.0025786 21998698 PMC3188575

[69] Kazmin D Nakaya HI Lee EK Johnson MJ van der Most R van den Berg RA . Systems analysis of protective immune responses to RTS,S malaria vaccination in humans. Proc Natl Acad Sci USA. (2017) 114:2425–30. doi: 10.1073/pnas.1621489114 28193898 PMC5338562

[70] Leroux-Roels I Davis MG Steenackers K Essink B Vandermeulen C Fogarty C . Safety and immunogenicity of a respiratory syncytial virus prefusion F (RSVPreF3) candidate vaccine in older adults: phase 1/2 randomized clinical trial. J Infect Dis. (2023) 227:761–72. doi: 10.1093/infdis/jiac327 35904987 PMC10044090

[71] Couch RB Bayas JM Caso C Mbawuike IN Lopez CN Claeys C . Superior antigen-specific CD4^+^ T-cell response with AS03-adjuvantation of a trivalent influenza vaccine in a randomised trial of adults aged 65 and older. BMC Infect Dis. (2014) 14:425. doi: 10.1186/1471-2334-14-425 25078387 PMC4138369

[72] Morinobu A Gadina M Strober W Visconti R Fornace A Montagna C . STAT4 serine phosphorylation is critical for IL-12-induced IFN-γ production but not for cell proliferation. Proc Natl Acad Sci USA. (2002) 99:12281–6. doi: 10.1073/pnas.182618999 12213961 PMC129436

[73] Gomez JA Wapinski OL Yang YW Bureau J-F Gopinath S Monack DM . The NeST long ncRNA controls microbial susceptibility and epigenetic activation of the interferon-γ locus. Cell. (2013) 152:743–54. doi: 10.1016/j.cell.2013.01.015 23415224 PMC3577098

[74] Loos C Coccia M Didierlaurent AM Essaghir A Fallon JK Lauffenburger D . Systems serology-based comparison of antibody effector functions induced by adjuvanted vaccines to guide vaccine design. NPJ Vaccines. (2023) 8:34. doi: 10.1038/s41541-023-00613-1 36890168 PMC9992919

[75] Budroni S Buricchi F Cavallone A Bourguignon P Caubet M Dewar V . Antibody avidity, persistence, and response to antigen recall: comparison of vaccine adjuvants. NPJ Vaccines. (2021) 6:78. doi: 10.1038/s41541-021-00337-0 34021167 PMC8140094

[76] Sahin U Muik A Vogler I Derhovanessian E Kranz LM Vormehr M . BNT162b2 vaccine induces neutralizing antibodies and poly-specific T cells in humans. Nature. (2021) 595:572–7. doi: 10.1038/s41586-021-03653-6 34044428

[77] Paila YD Pajon R Banbury B Fields P Maglinao M De Rosa SC . Potent and dose-sparing next-generation SARS-CoV-2 vaccine, mRNA-1283, induces polyfunctional and durable T cell immunity. NPJ Vaccines. (2026) 11:74. doi: 10.1038/s41541-026-01402-2 41714624 PMC13031613

